# Decreased *DGCR8* Expression and miRNA Dysregulation in Individuals with 22q11.2 Deletion Syndrome

**DOI:** 10.1371/journal.pone.0103884

**Published:** 2014-08-01

**Authors:** Chantal Sellier, Vicki J. Hwang, Ravi Dandekar, Blythe Durbin-Johnson, Nicolas Charlet-Berguerand, Bradley P. Ander, Frank R. Sharp, Kathleen Angkustsiri, Tony J. Simon, Flora Tassone

**Affiliations:** 1 Institute of Genetics and Molecular and Cellular Biology, University of Strasbourg, Strasbourg, France; 2 Department of Biochemistry and Molecular Medicine, University of California Davis, Sacramento, California, United States of America; 3 Department of Public Health Sciences, UC Davis Medical Center, Sacramento, California, United States of America; 4 MIND Institute, UC Davis Medical Center, Sacramento, California, United States of America; 5 Department of Neurology, UC Davis Medical Center, Sacramento, California, United States of America; 6 Department of Pediatrics, UC Davis Medical Center, Sacramento, California, United States of America; 7 Department of Psychiatry, UC Davis Medical Center, Sacramento, California, United States of America; NIDCR/NIH, United States of America

## Abstract

Deletion of the 1.5–3 Mb region of chromosome 22 at locus 11.2 gives rise to the chromosome 22q11.2 deletion syndrome (22q11DS), also known as DiGeorge and Velocardiofacial Syndromes. It is the most common micro-deletion disorder in humans and one of the most common multiple malformation syndromes. The syndrome is characterized by a broad phenotype, whose characterization has expanded considerably within the last decade and includes many associated findings such as craniofacial anomalies (40%), conotruncal defects of the heart (CHD; 70–80%), hypocalcemia (20–60%), and a range of neurocognitive anomalies with high risk of schizophrenia, all with a broad phenotypic variability. These phenotypic features are believed to be the result of a change in the copy number or dosage of the genes located in the deleted region. Despite this relatively clear genetic etiology, very little is known about which genes modulate phenotypic variations in humans or if they are due to combinatorial effects of reduced dosage of multiple genes acting in concert. Here, we report on decreased expression levels of genes within the deletion region of chromosome 22, including *DGCR8*, in peripheral leukocytes derived from individuals with 22q11DS compared to healthy controls. Furthermore, we found dysregulated miRNA expression in individuals with 22q11DS, including miR-150, miR-194 and miR-185. We postulate this to be related to *DGCR8* haploinsufficiency as *DGCR8* regulates miRNA biogenesis. Importantly we demonstrate that the level of some miRNAs correlates with brain measures, CHD and thyroid abnormalities, suggesting that the dysregulated miRNAs may contribute to these phenotypes and/or represent relevant blood biomarkers of the disease in individuals with 22q11DS.

## Introduction

22q11.2 Deletion Syndrome (22q11DS) (OMIM #611867), also known as DiGeorge (OMIM #188400) and Velocardiofacial (OMIM #192430) syndromes, derives from the most common chromosomal deletion associated with birth defects in humans and it is estimated to occur in 1∶4000 to 1∶9700 live births [1]–[5]. Deletions are thought to arise from the mis-pairing of low copy repeat (LCR) regions during cell division, resulting in a 3 Mb deletion in 70–80% of individuals (between LCRs A-D) or to a nested 1.5 Mb deletion in 15–30% of individuals (between LCRs A–B) [6], [7]. Atypical deletions within the 22q region are present in the remainder of individuals [6].

Individuals with 22q11DS are characterized by a wide range of clinical manifestations typically including abnormal development of a variety of organs and structures (heart, palate, thyroid and kidney), immunological anomalies, and neurological deficits that lead to behavioral disorders and learning disabilities. 22q11DS is also the second most common genetic cause of congenital heart defects (CHDs) and cardiovascular malformations are present in 75% of patients. Conotruncal cardiac defects are the most common type of CHDs in these individuals and include truncus arteriosus, Tetralogy of Fallot, interrupted aortic arch, pulmonary atresia, and ventricular septal defects. Additionally, 22q11DS is the leading genetic cause of cleft palate that affects up to 42% of individuals. Kidney defects are seen in up to one-third of these individuals and can require life-long medical attention [8]. A high prevalence of behavioral disorders has also been observed in individuals with 22q11DS. Rates of Attention Deficit Hyperactivity Disorder (ADHD) vary from 52% in preteens to around 15–30% across adulthood. Anxiety disorders are highly prevalent at 50–60% in childhood and somewhat less in adulthood [9]. Several studies based on parent reports have indicated rates of 20–50% of Autism Spectrum Disorders (ASDs) in children with 22q11DS although smaller studies using gold standard diagnostic methods found that no child met the criterion for ASD [10]–[12].

0.6–2% of schizophrenia cases have been attributed to the 22q11DS microdeletion and it is estimated that 30% of individuals with 22q11DS will develop some type of schizophrenia later in adolescence or adulthood [9], [13]–[15]. With respect to neural anomalies, individuals with 22q11DS show decreases in total and regional brain volumes [16], [17]. In particular, the hippocampus, which is involved in memory and spatial processing, has been strongly implicated in the pathophysiology of schizophrenia in the general population and in 22q11DS [14], [16], [18]–[20]. Hippocampal volume has also been linked to verbal IQ scores, which are reduced in individuals with 22q11DS and have been found to predict psychosis risk in that population [21]–[25].

Thirty genes are hemizygously deleted in the 1.5 Mb deletion and up to 60 genes are hemizygously deleted in the 3 Mb deletion [26]. However, it is not known if disease phenotypes arise from a combinatorial effect of reduced dosage from multiple genes that act together to lead to this diverse spectrum of phenotypes. The majority of genes located in the chromosome 22 deletion region have roles in RNA processing and signaling. Meechan et al. [27] demonstrated a significant decrease in mRNA expression levels for nine genes located within this region (*Prodh2*, *Zdhhc8*, *Comt-mb, Tbx1, Ufd1l, Hira, Idd, Ranbp1*, and *T10*) in the aortic arches and heart of LgDel mouse embryos. Additionally, gene expression levels of all genes located in the deletion region have been shown to be decreased in brains of a 22q11DS mouse model when compared to that of wild type (WT) mice [27]–[29].

Candidate genes for the CHD phenotype, such as *TBX1*, have been highly touted; however, no pathogenic mutations have been found [30]–[32]. Additionally, the presence of 22q11DS individuals with CHD but without a *TBX1* deletion suggests that *TBX1* alone does not lead to CHD [33].

One of the genes deleted in the majority of individuals with 22q11DS is the DiGeorge Critical Region Gene 8 (*DGCR8*) gene which encodes a crucial component of the microprocessor complex that contributes to microRNA (miRNA, miR) biogenesis and therefore, to global gene regulation. miRNAs are ∼22 nucleotide long and regulate gene expression, operating primarily through post-transcriptional gene silencing by binding to their target RNAs and are involved in many biological processes including development, cell death and cell metabolism [34]. In animals, the microprocessor complex processes mature miRNAs to target specific mRNAs for translational repression [35]. The *Dgcr8* ^+/−^ mice shows behavioral and cognitive anomalies including hyperactivity, abnormalities in sensorimotor gating, and impaired spatial working memory consistent with altered brain miRNA biogenesis due to *Dgcr8* haploinsufficiency [29]. Indeed, Stark et al. showed that prefrontal cortex and hippocampal miRNA levels were significantly downregulated (∼20–70%) and their mRNA target transcripts were shown to be significantly dysregulated in a Df16+/− mouse model [29]. Fenelon et al. [36] demonstrated that *Dgcr8^+/−^* mice have altered neuronal morphology and synaptic properties indicative of the altered short-term plasticity underlying cognitive dysfunction seen in individuals with 22q11DS. Muscle specific *Dgcr8* ^−/−^ mice demonstrated downregulation of a subset of mature cardiac enriched miRNAs, specifically miR-1, miR-133a, and miR-208, and experienced premature lethality within 2 months likely due to cardiac failure [37]. It has also been reported that loss of miRNAs in the neural crest (cells derived from form the pharyngeal arches in the developing embryo that eventually mature into the palate, face, and heart) lead to cardiac defects that are also observed in individuals with 22q11DS [38]. Lastly, a recent report demonstrated miRNA dysregulation in individuals with 22q11DS suggest a role in the immunological, cardiac and hypocalcemic phenotypes observed in this syndrome [39].

Here, we report on the decreased expression levels of several genes, including *DGCR8*, within the 22q deletion region in individuals with 22q11DS and compared to age matched controls. Furthermore, we report on miRNA dysregulation and on the potential contribution of specific miRNAs to multiple neurological and structural phenotypes seen in these individuals.

Our findings support the hypothesis that alteration of the miRNA landscape in individuals with 22q11DS contributes to the observed neuronal cell dysfunction and clinical phenotypes.

## Results

### Human Subjects

Demographic information and clinical data are shown in Table 1 for the subjects with 22q11DS. Clinical evaluation found that sixteen subjects (53%) presented with CHD (including Tetralogy of Fallot, atrial septal defects, polmonary atresia and ventricular septal defects), 33% had hypocalcemia, 10% has renal abnormalities, 16% had thyroid abnormalities and 20% had moderate or severe seizure (Table 1).

**Table 1:** Demographic and clinical data of the individuals with 22q11Ds included in the study.

Individual	Age	Gender	Deletionsize	Type ofCHD^a^	Hypocalcemia^b^	Seizures^d^	RenalAbn^b^	ThyroidAbn^b^	Left HPCVolume(mL)^e^	RightHPCVolume(mL)^e^	WholeBrainVolume(mL)^f^	HeadCircumference
1	9	F	3 Mb	0	2	0	0	0	1.63	1.88	1242.56	54.60
2	8	M	3 Mb	0	0	2	0	2	1.32	1.43	1070.68	48.5
3	15	M	3 Mb	0	0	0	0	0	1.75	1.97	1231.16	54.5
4	14	M	3 Mb	0	0	0	0	0	1.6	1.67	1353.01	59 (N/A)
5	8	M	3 Mb	0	0	0	0	0	1.67	1.75	1294.11	52
6	10	M	3 Mb	TOF, VaR	0	0	N/A^c^	0	1.29	1.35	1089.06	51.75 (N/A)
7	11	F	3 Mb	TA	2	0	2	2	0.9	0.95	1006.1	49.5
8	9	M	3 Mb	VR	0	0	0	0	1.47	1.63	1243.89	53
9	11	M	3 Mb	TOF, PA	2	0	0	0	N/A	N/A	1188.26	52.07
10	15	F	3 Mb	TOF, VSD	0	0	0	0	1.09	1.27	1084.95	N/A
11	12	F	3 Mb	VR, BAV	2	0	0	0	1.56	1.5	1234.23	57
12	8	M	3 Mb	0	0	0	0	0	1.41	1.27	1237.97	53.5
13	11	M	3 Mb	0	0	0	0	0	N/A	N/A	N/A	53.5
14	14	M	3 Mb	0	2	0	0	2	1.35	1.54	1200.58	55.2
15	5	M	3 Mb	0	0	0	0	0	N/A	N/A	N/A	55.5
16	4	F	3 Mb	TA	2	1 & 2	0	2	N/A	N/A	N/A	50.5
17	4	F	3 Mb	TA	2	1	0	2	N/A	N/A	1108.88	50.5
18	11	F	3 Mb	VSD	0	0	0	0	N/A	N/A	N/A	51.5
19	11	M	3 Mb	0	2	1	0	0	N/A	N/A	1432.14	57
20	6	M	3 Mb	ASD/VSD	0	0	0	0	N/A	N/A	1214.46	51.5
21	8	M	3 Mb	VR	0	2	2	0	N/A	N/A	N/A	56
22	8	M	3 Mb	0	0	0	2	0	N/A	N/A	N/A	52.5
23	8	M	3 Mb	TOF	2	0	0	0	N/A	N/A	N/A	54.6
24	11	F	3 Mb	PDA, ASD/VSD, PFO	0	0	0	0	N/A	N/A	N/A	53.5
25	5	F	3 Mb	TOF	0	0	0	0	N/A	N/A	N/A	52.07
26	4	F	3 Mb	0	N/A^c^	N/A	N/A	N/A	N/A	N/A	N/A	N/A
27	5	M	3 Mb	0	0	0	N/A	N/A	N/A	N/A	N/A	51
28	8	M	3 Mb	0	0	0	0	0	1.54	1.66	1279.48	54.6
29	3	F	3 Mb	ASD/VSD	0	0	0	0	1.21	1.61	9572.97	48.5
30	9	M	1.5 Mb	IAA, ASD/VSD, mild aortic stenosis	2	1 & 2	0	0	1.78	1.91	1206.48	53

a TOF = Tetralogy of Fallot; PA = Pulmonary Atresia; VSD = Ventricular Septal Defect; TA = Truncus Arteriosus; VR = Vascular Ring; BAV = Bicuspid Aortic Valve, VaR = Valve Replacement; ASD = Atrial Septal Defect; AAA = Aortic Arch Abnormalities; IAA = Interrupted aortic arch.

b 0 = No; 2 = Yes.

c N/A = Data not available.

d 0 = No; 1 = Hypocalcemic seizures; 2 = Yes.

e Average hippocampal volume for TD individuals is 2 mL.

f Average whole brain volume for TD individuals is 1200 mL.

HPC = hippocampus.

Abn = abnormality.

### Size Deletion Analysis

**Figure 1:**
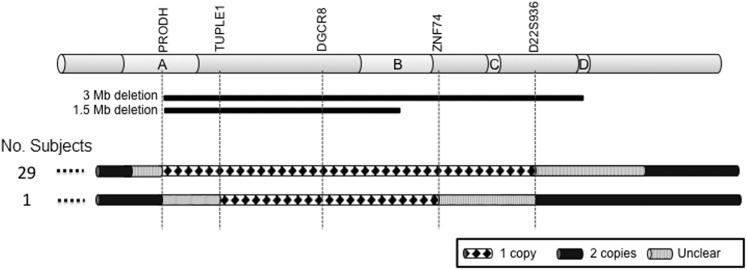
Diagram of the 22q11.2 deletion region. Schematic overview of chromosome 22 and the deletion endpoints characterized in the participants of this study using qPCR. Individuals 1 to 29 had a deletion between *PRODH* and *D22S936*. One individual had the deletion located between the *TUPLE1* and *ZNF74* genes. Gene used, order of genes, LCRs A–D, and the common 3 Mb and 1.5 Mb deletion are indicated. Thin black lines indicate deleted region, solid black thick lines indicate chromosomal regions present in two copies, and gray boxes indicate uncertain location of the deletion breakpoints. Each numbered chromosome represents one individual.

Deletion endpoints were verified using quantitative PCR (qPCR) in 30 individuals with 22q11DS. 29 individuals had the typical ∼3 Mb deletion spanning *PRODH*-*D22S936* while one individual had the smaller ∼1.5 Mb deletion spanning from *TUPLE1-ZNF74.* A diagram depicting the deletion endpoints observed in the individuals included in this study is shown in **Figure 1**. qPCR values are shown in **Table S1**. Interestingly, the individual with the ∼1.5 Mb deletion had an IQ = 87 but presented with severe symptoms, including multiple heart problems, ADHD, hypocalcemia and seizures (**Table 1**), supporting the hypothesis of a minimal critical region for disease located between LCR A-LCR B.

### mRNA expression levels of genes mapping within the deleted region

To determine if the hemizygous deletion resulted in reduced expression of genes mapping within the deleted region, we measured the gene expression levels in peripheral blood leukocytes derived from individuals with 22q11DS of the following six genes: catechol-O-methyltransferase; *COMT*, DiGeorge syndrome critical region gene 6; *DGCR6*, DiGeorge syndrome critical region gene 8; *DGCR8*, zinc finger DHHC-type containing 8; *ZDHHC8*, thioredoxin reductase 2; *TXNRD2*, and solute carrier family 25 member 1; *SLC25A1* and of one gene, the Glyceraldehyde 3-phosphate dehydrogenase (GAPDH) used as control. *COMT* encodes for a cytosolic enzyme that acts to degrade neuroactive monoamines such as dopamine and it has been considered a possible candidate gene for schizophrenia and other psychiatric disorders [40], [41]. *DGCR6* encodes for a putative gonadal protein that is involved with germ cell and gonadal development [42], [43]. *ZDHHC8* encodes for the palmitoyltransferase enzyme that is responsible for posttranslational modifications [44]. *ZDHHC8* has been associated with susceptibility to schizophrenia and Mukai et al. demonstrated that transfection of a *ZDHHC8* carrying construct into primary hippocampal neurons of *Df(16)A* ^+/−^ mice could recover dendritic spine density and growth to nearly WT levels [45]. *SLC25A1* and *TXNRD2* encode for mitochondrial proteins responsible for transporting citrate across the mitochondrial inner membrane and a thioredoxin reductase that plays a large role in intracellular redox regulation, respectively [46], [47].

**Figure 2:**
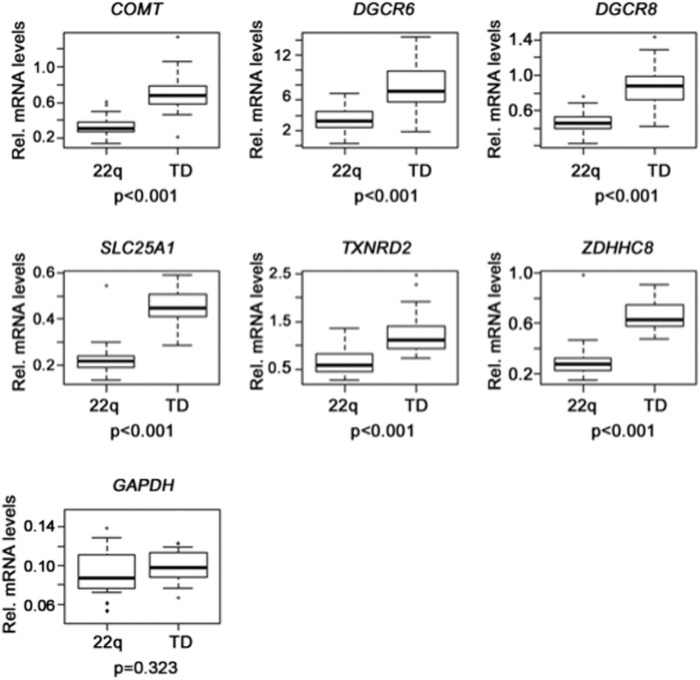
Gene expression levels. Box plots showing the relative transcript expression levels for six genes mapping within the deleted region and of the *GAPDH* gene, used as control. Total RNA was isolated from 90 individuals with 22q11DS (22q) and from 40 typical developing age-matched controls (TD). Error bars indicate standard errors. mRNA levels were normalized to the reference Glucuronidase gene. Respective p-values are shown.

**Figure 3:**
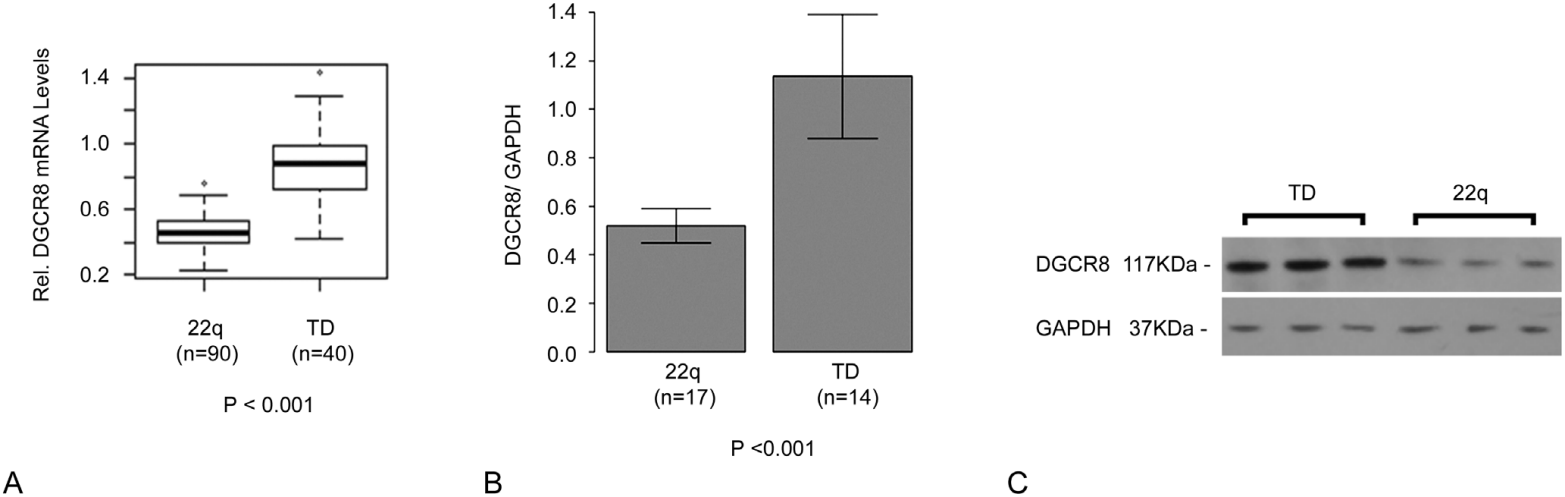
*DGCR8* expression levels. a) box plots showing a decreased *DGCR8* mRNA levels in subjects with 22q11DS compared to TD and b) DGCR8 protein expression levels measured by Western blot analysis shows a significant decrease in 22q11DS individuals when compared to TD controls. c) Representative Western blot showing decreased DGCR8 expression.

mRNA expression levels for each gene were measured in 90 individuals with 22q11DS and 40 age matched controls by quantitative real-time PCR (qRT-PCR). As expected, mRNA expression analysis showed ∼0.5 fold decrease in expression levels in peripheral blood leukocytes of those with 22q11DS when compared to controls for all six genes (**Figure 2**), although with some variation in levels**.** In addition to reduced mRNA expression levels, *DGCR8* protein expression levels, as measured by Western blot analysis, were also reduced in individuals with 22q11DS (**Figure 3**). As expected, no differences in GAPDH expression levels were observed between 22q11.2DS and healthy controls (**Figure 2**).

### miRNA Expression Levels

**Figure 4:**
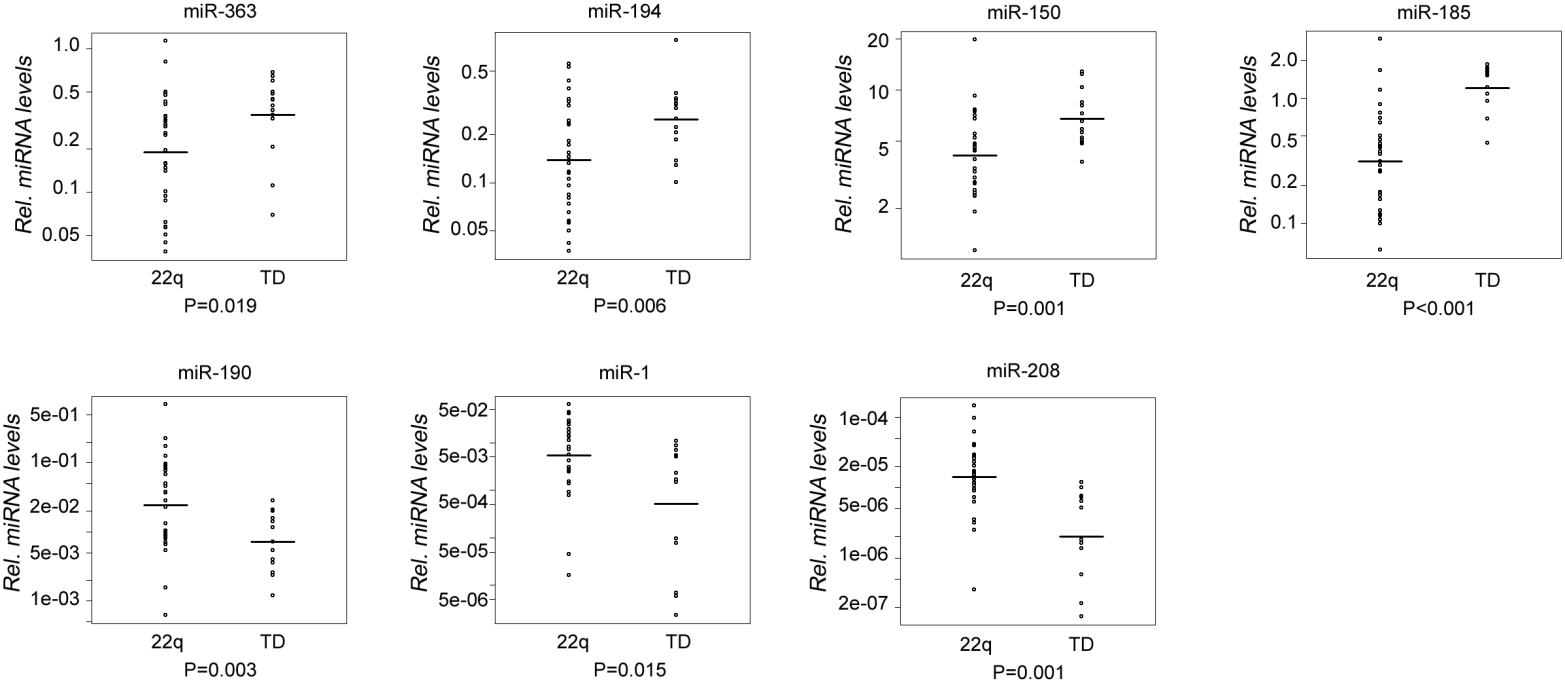
Altered miRNA expression in 22q11DS. Relative miRNA levels are shown for 7 miRNAs that demonstrated differential expression levels when comparing 30 individuals with 22q11DS and 15 TD individuals. miRNA levels were normalized to the reference U6 snRNA. Circles represent observed data. Horizontal lines represent geometric means. Respective p-values are shown.

To investigate the presence of miRNA dysregulation as a result of haploinsufficiency of the *DGCR8* gene, we measured the expression of selected miRNAs in 45 individuals, 30 with 22q11DS (same as those analyzed for deletion endpoints) and 15 typical developing control subjects (TD). The rationale for miRNA selection was based on their role in CHD and to their association with 22q11DS phenotypes observed in previous studies [29], [39]. Seven miRNAs showed a differential expression between the two groups (**Figure 4**). Of the differentially expressed miRNAs, miR-185, miR-150, miR-194, and miR-363 were downregulated in individuals with 22q11DS as compared to TD controls and miR-208, miR-190, and miR-1 were upregulated. We also tested the expression of two mirtrons, mi-877 and mi-1224, which are miRNAs processed by the splicing machinery, and whose biogenesis is therefore independent of DGCR8/DROSHA [48]. qRT-PCR analysis showed that no significant differences in expression levels of mi-877 and mi-1224 (p = 0.843 and p = 0.491, respectively) were present between the two groups (22q11DS, n = 30; TD, n = 15) suggesting that mono-allelic expression of *DGCR8* specifically reduced the activity of DROSHA, without affecting the splicing or the RNA polymerase II machinery. Additionally, we measured the expression level of primary-miR-324-5p and primary-miR-23b transcripts in the 30 individuals with 22q11DS and found that they were significantly increased (p<0.001 and p = 0.002 respectively) compared to those measured in 15 TD individuals, while the expression level of the mature miR-324-5p and miR-23b transcripts were decreased (p = 0.001 and p = 0.078 respectively) compared to those measured in TD **individuals**, strongly suggesting that the observed reduced expression levels of several miRNA are likely due to haploinsufficiency of DGCR8.

We then examined patient phenotypes to determine if the levels of specific miRNA(s) were associated with clinical phenotypes including brain volume, thyroid dysfunction, hypocalcemia, seizure and CHD.

**Figure 5:**
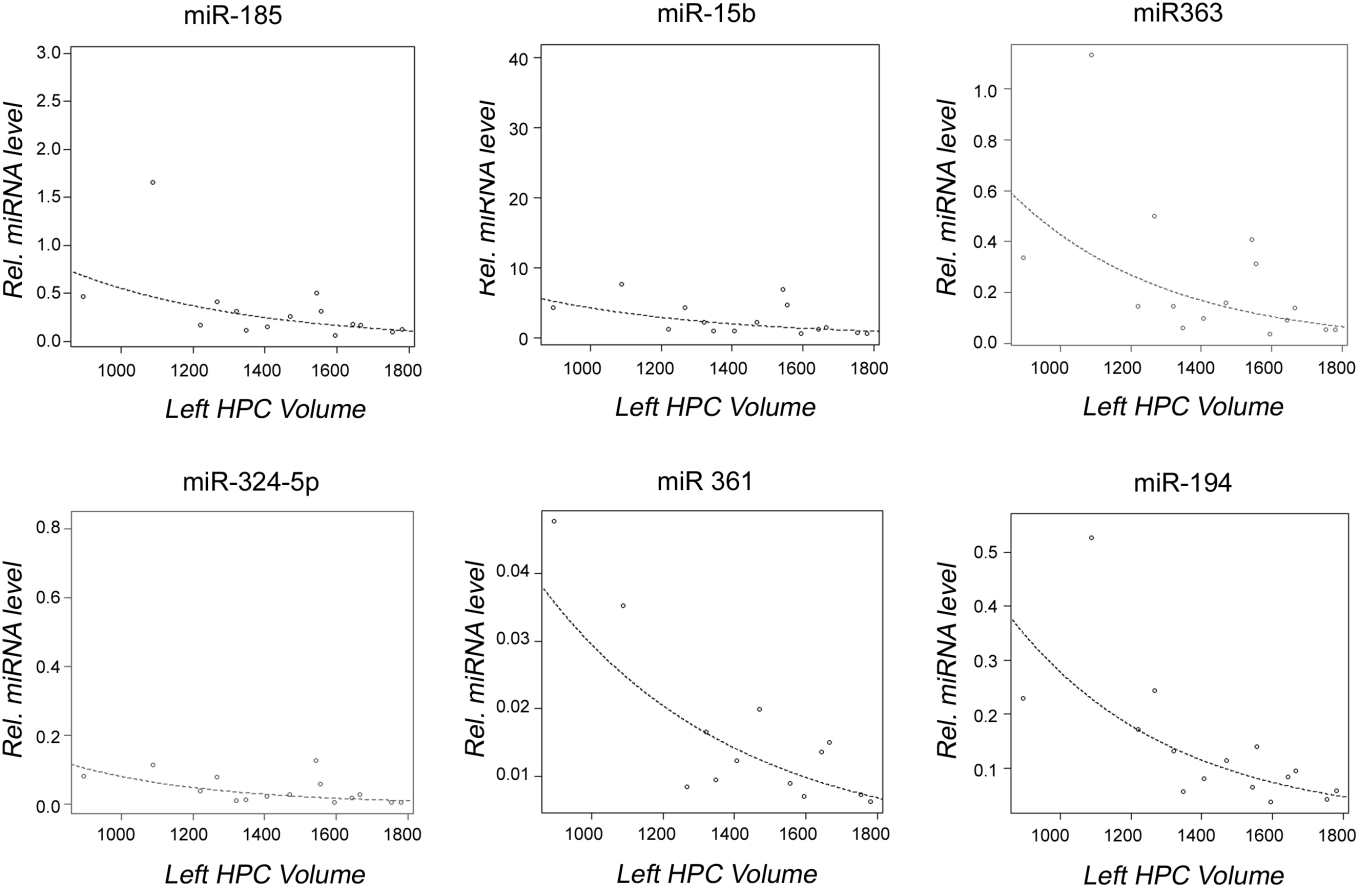
Observed correlation between dysregulated miRNAs and left hippocampal volume. Graphs showing a correlation between miRNA expression levels and left hippocampal volume within the 22q11DS group. Open circles represent individuals with 22q11DS; the line represents line of best fit. P-values given are for correlations.

**Figure 6:**
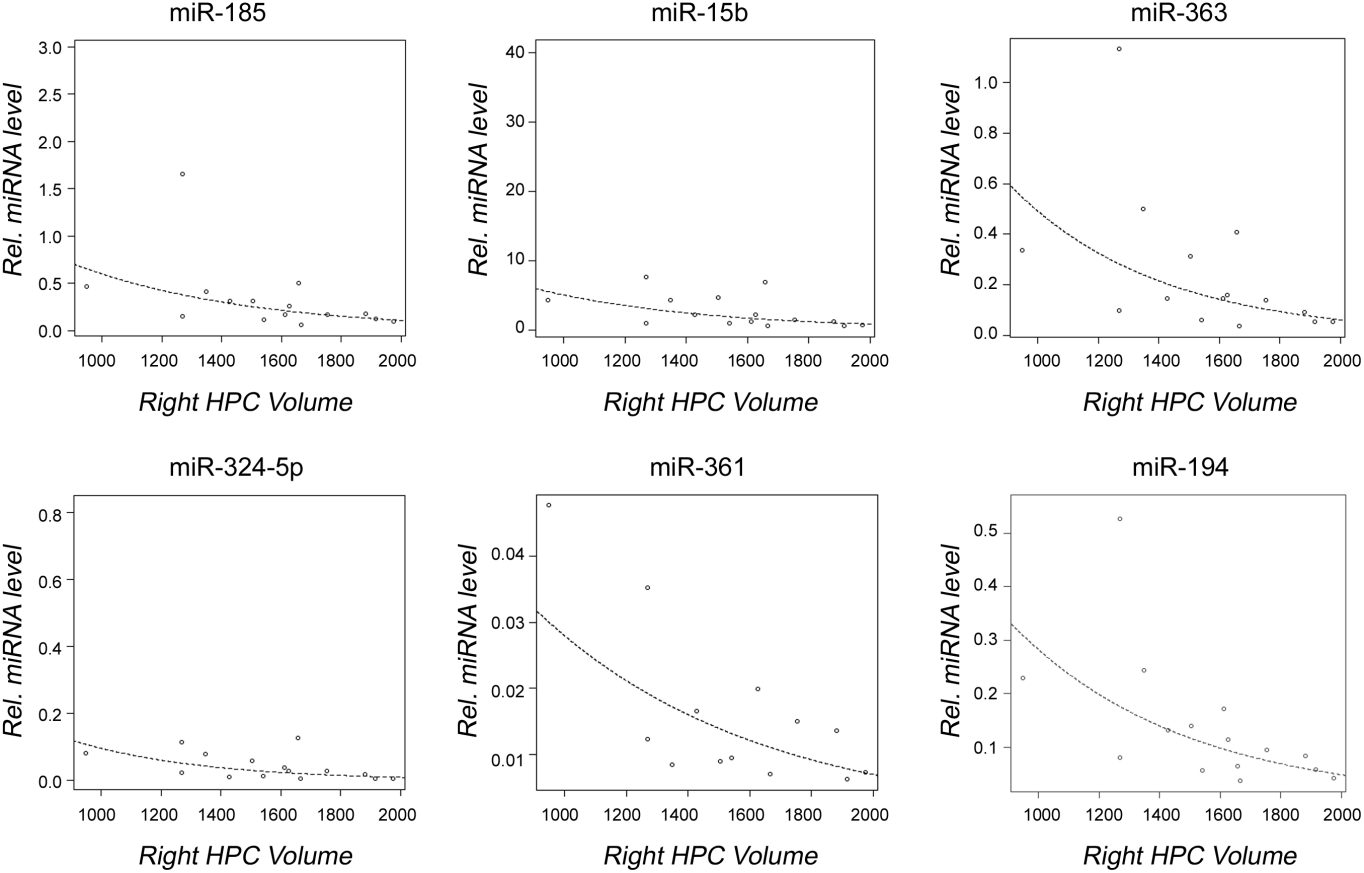
Observed correlation between dysregulated miRNAs and right hippocampal volume. Graphs showing a significant correlation between miRNA expression levels and right hippocampal volume within the individuals with 22q11DS are shown. Open circles represent individuals with 22q11DS; the line represents line of best fit. P-values given are for each miRNA.

**Figure 7:**
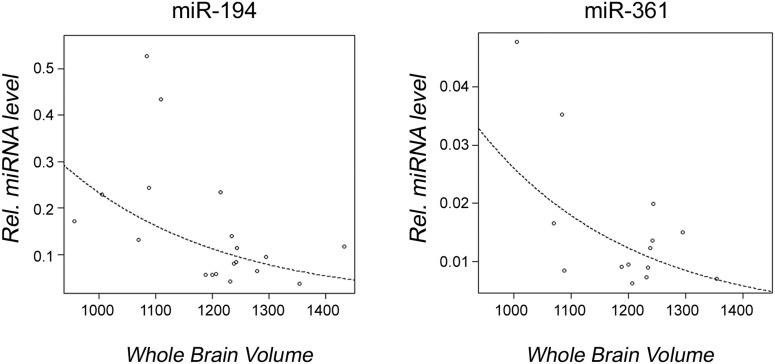
Observed correlation between dysregulated miRNAs and whole brain volume. Graphs showing a significant correlation between miRNA expression levels and whole brain volume within the individuals with 22q11DS are shown. Open circles represent individuals with 22q11DS; the line represents line of best fit. P-values given are for each miRNA.

A significant inverse correlation between decreased miRNA expression and increased brain volume was detected in the 22q11DS cohort. Specifically, six miRNAs (miR-185, miR-15b-3p, miR-363, miR-324-5p, miR-361-5p, and miR-194) were dysregulated in individuals with 22q11DS when examining left hippocampal volume (**Figure 5**), and also with right hippocampal volume (**Figure 6**), while the expression level of two miRNAs (miR-361-5p, and miR-194) significantly decreased with increased whole brain volume (**Figure 7**). In addition, our data showed that miR-194 was significantly up-regulated by 2.13 fold in individuals with 22q11DS who had thyroid dysfunction (p = 0.037), and by 2.02-fold in individuals with 22q11DS who had CHD (p = 0.012), compared to unaffected subjects. No significant differences in expression levels of any of the miRNAs analyzed were found when individuals with 22q11DS were compared to controls for seizures, renal abnormalities, head circumference and hypocalcemia.

## Discussion

Mammalian development is sensitive to perturbation of gene signaling such that either gain or loss of function of a single allele of genes mapping within the deleted region can affect development and function. However, additional factors can play a role and account for the broad variation in the phenotypes we observed in individuals with 22q11DS. It is not clear if altered gene dosage of a number of genes, or if any specific ones, lead to the observed phenotypes by means of a cumulative effect, or if other mechanisms play a role. The impact of several deleted genes is what likely may determine the overall 22q11DS phenotype and it has become increasingly clear that the 22q11DS symptoms most likely arise from a combination of factors including gene expression of genes inside and outside of the deletion region (modifier genes) and/or from the individual’s genetic background [49]. Inconsistent phenotypes have been reported in siblings and monozygotic twins that have concordant 22q deletions reflecting environmental effects [50]–[53]. We characterized the deletion endpoints of the subjects included in our study and found that the majority of individuals (n = 29) had the same 3 Mb deletion; however, one individual had a smaller, nested deletion spanning from *TUPLE1* to the *ZNF74* gene. Individuals with interstitial deletions and severe phenotype have been previously reported in the literature. McQuade et al. [54] described a patient with a 750 Kb deletion encompassing *COMT* and *TBX1.* He presented with many of the typical 22q11DS symptoms such as cleft palate, facial features typical for 22q11DS, low IQ, schizophrenia, OCD, and developmental delay. Individuals carrying an atypical deletion and yet presenting with the characteristic features of 22q11DS, suggest that a minimal critical region is sufficient to lead to disease phenotypes.

Altered dosage of several genes in the 22q deletion region is hypothesized to correlate with phenotypic variation. In the present study, we demonstrated approximately a 50% reduction in the expression levels of several candidate genes in the deleted region (*COMT*, *DGCR6*, *DGCR8*, *ZDHHC8*, *TXNRD2* and *SLC25A1*) in peripheral blood leukocytes from young and adolescent participants with 22q11DS, and demonstrate a ∼50% decrease in mRNA levels when compared to controls. One of these genes, *DGCR8*, plays an important role in miRNA biogenesis by encoding for a crucial component of the microprocessor complex that processes primary microRNA (pri-miRNAs) transcripts to mature microRNAs [29], [53].

Indeed, as a consequence of DGCR8 haploinsufficiency and as observed in the 22q deletion mouse model, we demonstrate miRNA dysregulation in peripheral blood leukocytes derived from individuals with 22q11DS [29], [55]. The lack of significant difference observed between individuals with 22q11DS compared to TD in the expression levels of two mirtrons, whose biogenesis depends on alternative, non-canonical, miRNA biogenesis pathways via splicing, in addition to higher expression levels of primary miRNA and of lower of mature miRNA of those found to be dysregulated in individuals with 22q11DS compared to TD, are supportive of the hypothesis that altered miRNA expression pattern observed in this study likely results from the hemizygous *DGCR8* expression [56]. These results also show that transcription and processing of miRNAs are not globally altered in 22q11DS and provide compelling evidence implicating microRNA-mediated dysregulation in 22q11DS.

Our findings confirm previous reports [29], [39], [55] and further suggest that haploinsufficiency of *DGCR8* could alter the miRNA expression landscape and contribute to the wide clinical phenotype, including cognitive, neurocognitive, psychiatric disorders and cardiac disease, observed in 22q11DS. Importantly, we demonstrate, for the first time, that altered miRNA levels correlates with brain measures which is intriguing in light of the recent report of hippocampus-dependent spatial learning, memory, and social behavior deficits and decreased hippocampal adult neurogenesis observed in the Dgcr8/mouse model [57].

**Figure 8:**
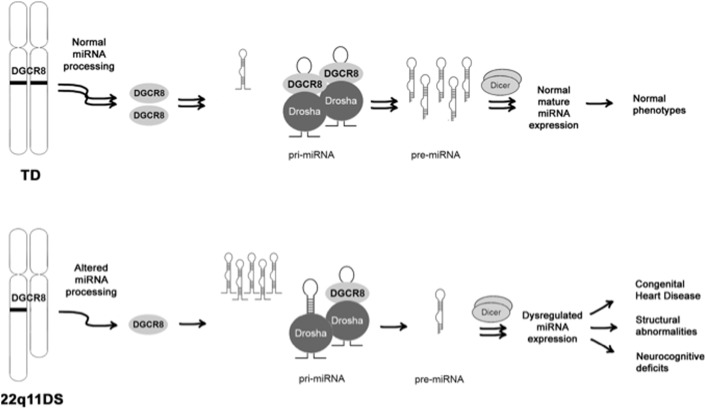
Proposed model of miRNA dysregulation in 22q11DS. Decreased levels of free *DGCR8* results in dysregulated expression of mature miRNAs, ultimately leading to development of the clinical phenotypes associated with 22q11DS.

Thus, we propose a model where hemizygous deletion of *DGCR8* leads to a decrease in microprocessor efficiency and to a dysregulation of miRNA expression, which ultimately contributes to the clinical phenotypes seen in individuals with 22q11DS (**Figure 8**).

miRNAs play an important role in the regulation and modulation of many biological functions and their altered expression has been observed in a variety of human diseases including cardiovascular and heart diseases [58].

A number of miRNAs including miR-194, miR-361, miR-150 and miR-185 were found to be dysregulated in 22q11DS. In particular, highly significant lower expression levels were observed for miR-194, miR-361 and miR-185 in individuals with 22q11DS across all of our phenotypic neuronal measures. Down-regulation of miR-194 was observed in both the prefrontal cortex and hippocampus in the 22q11DS mouse model, suggests a potential role in the development of the central nervous system [29]. miR-194 is also highly enriched in kidney, differentially expressed in renal carcinoma and can affect cell migration [59]. Renal abnormalities are often observed in 22q11DS; however they were present only in a few individuals in our group (Table 1). More importantly, we observed a statistically significant difference in its expression levels within the 22q11 group between subjects with CHD compared to those without, particularly in relation to the recent findings of miR-194 involvement in acute myocardial infarction in individuals experiencing heart failure [60].

The observed down-regulation of miR-185 was expected as it is located within the deleted region of chromosome 22 and it was found to be significantly down-regulated across many of the neurological measures. miR-185 is implicated in many neurological disorders leading to hypotonic infants and it has also been suggested as a key player in neuronal development. In this study down-regulation of miR-185 correlated with brain volume, in agreement with findings in both the prefrontal cortex and the hippocampus of the mouse model of 22q11DS [29]. miR-185 contributes to dendritic and spine development deficits in hippocampus of the Df(16)A+/− mouse model [55]. This and previous research showing the presence of miR-185 at the synapses [61], [62], suggest that miR-185 may have a role in neural function and constitute a key gene regulator in 22q11DS.

In this study we also observed that decreased miR-324-5p expression levels correlated with increased left and right hippocampal volume; interestingly miR-324-5p promotes neuronal differentiation and it may contributes to the development of defined neuronal subtypes [63], [64].

Importantly the miRNAs, which expression was found altered in this study, are expressed in both peripheral blood leukocytes and brain tissue and for several of them the expression was found to be altered in psychiatric disorders including schizophrenia and depression [65]–[68].

Several miRNAs have been implicated in cardiogenesis and heart development including miR-1, miR208 and miR-190 [69]–[71]. In the presented study we observed an increased expression of miR-208 and miR-190 in 22q11DS compared to controls; interestingly a significant up-regulation of these miRNAs were reported in patients with myocardial infarction [69] suggesting their contributing role in cardiac disease. Thus, it is possible that upregulation of these miRNA in patients with 22q11DS is secondary to cardiac alterations and likely overcome the decrease processing efficiency due to DGCR8 haploinsufficiency.

Finally, we demonstrate that several miRNAs including miR-361 and miR 194 are downregulated in blood, suggesting that dysregulation of the miRNA biogenesis in 22q11DS occurs in blood and that the degree of dysregulation can correlate with psychiatric, neurocognitive and immunological features of 22q11DS. Several reports have indicated the usefulness of using blood for miRNA expression studies and have demonstrated altered expression of blood and brain miRNAs involved in brain plasticity and maturation associated to schizophrenia and ASD [72]–[75]. These studies suggest that lymphocytes could reflect, or partially share, the molecular phenotype of neural cells and could therefore be used in studies of psychiatric disorders. This is important since the identification of altered miRNAs expressed in blood could be used as biomarkers to identify potential therapeutic targets and to monitor the response to potential therapeutic approaches of the disease.

We expected that some miRNAs, including miR-1 and miR-208, although differentially expressed in subjects with 22q11DS compared to TD, were dysregulated between individuals with 22q11DS with CHD compared to those without CHD, but we did not detect any difference in levels between the two groups. This may reflect the age of the individuals studied as previous studies indicating a critical role for microRNAs in maintaining cardiac function were performed on prenatal mice [37]. The observed lack of association could also be due to the modest number of 22q11DS subjects with specific types of CHD, which is one of the limitations of this study.

Elucidating miRNA dysregulation in 22q11DS will promote understanding of the contribution of miRNAs in neurocognitive, cardiac, behavioral and other common diseases that demonstrate differential miRNA expression such as cancer. While it is well known that individual miRNAs have important roles in neuronal and cardiovascular function and development, the *in vivo* implications are less well understood. Here, we demonstrate that miRNAs are associated with a variety of clinical abnormalities and importantly, that they can be detected in blood suggesting a potential use for circulating miRNAs as non-invasive biomarkers. That circulating miRNA can serve as novel diagnostic markers and that these miRNAs can be delivered to recipient cells, where they can regulate translation of target genes, has been proposed for a wide range of cardiovascular disease [76].

The documentation of molecular markers or abnormalities that underlie the phenotypic involvement of psychiatric, cardiac, or neurological dysfunction will lead to earlier treatment for the problems seen in childhood and adolescence in individuals with 22q11DS.

Our findings, in addition to several studies in the mouse models of 22q11DS, provide compelling evidence that the syndrome results in abnormal processing of miRNAs. Our findings are in agreement with those recently reported by de la Morena et al. but extended into a wider age range [39]. We also reported correlations between miRNA dysregulation and key phenotypic characteristics of 22q11DS including those involving the CNS and thyroid abnormalities and more importantly demonstrated an effect of decreased miRNA levels in brain measures.

In conclusion, we show that miRNA dysregulation in 22q11DS is due to *DGCR8* haploinsufficiency and that this may contribute to significant dysregulation of their target mRNAs ultimately leading to the clinical phenotype observed in these individuals. However, it remains to be determined if the miRNA dysregulation here described is also present in brain tissue and therefore playing a key role in the neurological phenotype seen in 22q11DS. Further studies are also necessary to determine if miRNA profiles along with other biomarkers and cognitive testing could potentially provide a more comprehensive and early diagnosis of 22q11DS.

Finally, the ultimate challenge will be to identify and validate the target genes that are affected by those microRNAs that are found dysregulated in 22q11DS and to characterize the pathways of involvement in a much more comprehensive manner in order to improve our understanding of how alterations in microRNA-mediated genetic networks can contribute to the pathophysiology of 22q11DS. Although further studies are required to assess the mechanism of action of miRNA acting as gene modulators, ultimately this information will be of relevance for the developing of novel therapeutic strategies.

## Materials and Methods

### Human Participants

Participants were recruited for behavioral and developmental assessments at the UC Davis Medical Investigation of Neurodevelopmental Disorders (MIND) Institute located in Sacramento, California, under written consent from the next of kin, caretakers, or guardians on the behalf of the minors/children participants and according to a UC Davis Institutional Review Board (IRB) approved protocol. The diagnosis of 22q11DS was performed by FISH analysis using the *TUPLE1* probe. Age- and gender-matched typically developing children (TD) were used as controls and the inclusion criteria was FSIQ >85, normal ADHD and ADS scores [77] and normal social functioning [78]. All participants were examined by a developmental behavioral pediatrician or child and adolescent psychiatrist. Physical conditions (cardiac, neurologic, immunologic, renal, etc.) were obtained from the parents and when available, medical record abstraction. Medical conditions were rated on a severity scale described in Table 1. Molecular measures were determined based on sample availability. Peripheral blood was collected in EDTA blood collection tubes and in Tempus tubes (Applied Biosystems, Foster City, CA) and DNA and totRNA respectively isolated for further analysis. For mRNA expression analysis, we examined a total of 130 individuals, 90 individuals with 22q11DS including 40 males and 50 females, known to have 22q11DS by FISH and, 40 TD individuals including 19 males and 21 females. Age range of participants was 7 to 21 years (mean age is 13.1±3.5 years for TD and 10.9±2.6 years for 22q11DS individuals). For protein expression analysis, we examined a total of 31 individuals (17 22q11DS, including 14 males and 3 females, and 14 TD, including 5 males and 9 females) where the age range of participants is 7–15 years (mean age is 11.4±2.6 (22q) and 10.5±2.4 (TD)). In a subgroup of them (30 individuals with 22q11DS), miRNA analysis and deletion endpoint analyses were both carried out. Specifically, for miRNA expression analyses, we examined a total of 45 individuals (30 22q11DS, including 19 males and 11 females, and 15 TD, including 9 males and 6 females), where the age range of participants was 4–15 years and the mean age is 10.7±4.5 (TD) and 8.9±3.4 (22q11DS) years for which totRNA (from PAX tubes; Qiagen, Valencia, CA) containing miRNAs was available. For deletion endpoint analyses, we examined a total of 30 individuals with 22q11DS, including 19 males and 11 females where the age range is 4–15 years (mean 8.9±3.4 years). There was no statistical difference in age range between the two groups for any of the analyses.

### Deletion Endpoint Analysis by qPCR

qPCR and quantification of deletion endpoints was analyzed using methods described in Weksberg et al. [79] with the following minor modifications. Samples from 30 subjects with 22q11DS (19 male and 11 female) and 9 typically developing (TD) subjects (16 male and 8 female) were analyzed. Primers were designed using Primer Express v2.0 (Applied Biosystems, Carlsbad, CA) and followed the recommended primer design guidelines. Target assays, *D22S181, DGCR6, PRODH, TUPLE1, ZNF74, LZTR1, D22S936*, and *VPREB1*, and reference assays, *HEM3* and *G6PDH*, were used as described in Weksberg et al. [79]. The sequences of additional primers used including *TBX1, DGCR8, SCARF2*, and *SHGC-2421* were as follow: *Tbx1-F:* GTCCGAAATCAAGCGAGTGAGTA; *Tbx1*-*R:* GTGGAGGAAACCCTGGTCAAC; *DGCR8-F:* GCATGTGTTCCTTCTGCTCTGAT; *DGCR8*-*R:* CAGGACGCACTGAGGAGGTAGT; *SCARF2*-*F:* CATGGTGTAGGGCCAGTCTATCC; *SCARF2*-*R:* CAGCCATTCGCACTTTAGAGAAA; *Shgc*-*2421-F:* TCATGTGGGTGCTGGTACAT; *Shgc*-*2421-R*: AGCTTCAGGCTCTCCAGACA.

Reactions were performed using FastStart Universal SYBR Green Master Mix (Roche, Applied Science, Indianapolis, IN) (which includes the internal reference (ROX)), forward and reverse primers at final concentrations of 800 nM for the 22q11DS target primers and 400 nM for the reference primers, and 10 ng of genomic DNA. The qPCR reactions were run using the Applied Biosystems 7900HT FAST real-time PCR system with 2 min at 50°C, 10 min at 95°C followed by 40 cycles of 15 sec at 95°C and 60 sec at 60°C. qPCR data was analyzed using the comparative Ct method after data normalization [79].

### Quantitative Real Time-PCR for gene expression analysis of genes in the deleted region

Total RNA was isolated from Tempus tubes (Applied Biosystems, Carlsbad, CA) from blood drawn from 130 individuals, 90 with 22q11DS (22q) and 40 TD individuals using standard procedures. RNA was quantified with a NanoDrop 1000 UV/VIS spectrophotometer (Thermo Fisher Scientific, Inc., Waltham, MA). mRNA expression levels were measured using a quantitative-fluorescence reverse transcription polymerase chain reaction (qRT-PCR) method and analyzed on a 7900HT FAST real-time PCR system (Applied Biosystems, Carlsbad, CA). cDNA synthesis and real-time PCR were performed following previous methods [80]. Assay on Demand Gene Expression assays (Applied Biosystems, Carlsbad, CA) were used for each gene.

### Western Blot

Lymphocytes pellets isolated from 2 ml whole blood and available from 31 individuals (17 22q11DS and 14 TD) were washed twice using a PBS (137 mmol/L NaCl, 2.7 mmol/L KCl, 4.3 mmol/L Na_2_KH_2_PO_4_, pH 7.4) (Gibco, Carlsbad, CA) and Calbiochem protease inhibitor cocktail III (Millipore, Billerica, MA) and then resuspended in MPER lysis buffer (Pierce, Rockford, IL) with Roche complete protease inhibitor tablet (Roche Diagnostics, Indianapolis, IN) and Calbiochem cocktail III. Cell lysis was performed by sonication using the LabQuake (Thermo Fisher Scientific, Waltham, MA). The Quick Start Bovine Serum Albumin Standard set kit (BioRad Laboratories, Hercules, CA) was used according to manufacturer’s instructions for quantification of protein concentration. Cell-extracted proteins were separated using electrophoresis at 80 V, at RT, on a 8–13% Criterion Tris-HCl gel (BioRad Laboratories, Hercules, CA) in running buffer (25 mmol/L Tris, 192 mmol/L glycine, 0.1% SDS, pH 8.3). Proteins were transferred for 3 hours at 250 mA, at room temperature, using Criterion Cell Blotter System (BioRad, Hercules, CA) onto nitrocellulose membranes (BioRad Laboratories, Hercules, CA) in 25 mmol/L Tris, 192 mmol/L glycine, and 20% methanol. Ponceau staining of the nitrocellulose membrane and Coommassie Blue (Invitrogen, Carlsbad, CA) staining of the gel verified efficient transfer of proteins. Following the transfer, membranes were blocked with 5% nonfat dry milk in T-TBS (100 mmol/L Tris, 150 mmol/L NaCl, 0.1% polyoxyethylene sorbitan monolaurate), and then incubated with secondary antibody (1∶20,000 mouse anti-GAPDH (Millipore, Billerica, MA) and 1∶400 rabbit anti-DGCR8 (Proteintech, Chicago, IL). Super Signal West Dura substrate (Pierce, Rockford, IL) was used for the detection of antibodies. AlphaView gel analysis software was used to analyze band intensities (Protein Simple, Santa Clara, CA).

### miRNA Extraction, RT-PCR and target analysis

Total RNA from 45 individuals (30 22q11DS and 15 TD) was isolated from PAX tubes (Qiaen, Valencia, CA) and/or from cell pellets using Trizol (Life Technologies, Carlsbad, CA). cDNAs were generated using the miScript II RT Kit (Qiagen, Valencia, CA) for quantification of miRNAs or the Transcriptor High Fidelity cDNA Synthesis Kit (Roche Diagnostics, Indianapolis, IN) for quantification of mRNAs. qPCR of miRNAs was performed using the miScript Primer Assay (Qiagen, Valencia, CA) and miScript Sybr Green PCR Kit (Qiagen, Valencia, CA) in a LightCycler 480 (Roche Diagnostics, Indianapolis, IN) with 15 min at 94°C followed by 50 cycles of 15 s at 94°C, 20 s at 55°C, and 20 s at 72°C. U6 snRNA was used as standard.

### Statistical Analysis

For mRNA and protein expression, a two-sample t-test was used to test for differences in mean expression between the 22q11DS and TD groups for each gene. P-values were adjusted using the Bonferroni correction. Two sample t-tests were used to compare miRNA expression between TD and all 22q11DS participants, or between 22q11DS participants with and without a given clinical phenotype. miRNA expression was compared using one-way ANOVA followed by Tukey HSD pairwise comparisons between TD participants, those with 22q11DS and CHD, and those with 22q11DS but without CHD. miRNA expression was correlated with brain measurements using linear regression. miRNA data were log transformed prior to analysis; results are reported on the original scale of the data as fold changes. Analyses were conducted using R, version 3.0.0 (R Core Team, 2013).

## Supporting Information

Table S1 **Fold copy number change by qPCR.** (ΔKC_t_) for individuals with 22q11DS (n = 30) and TD individuals (n = 3) for three assays (PRODH, DGCR8, and D22S936) are shown. ΔKC_t_ values for TUPLE1 and ZNF74 for one individual (#30) with an atypical deletion are shown to better characterize the deletion size. ΔKC_t_ values of 0±0.35 indicate an equal ratio of the target and reference (normal) while values of −1±0.35 indicate a hemizygous deletion.
(DOCX)
