# Supplementary material for: Decreased DGCR8 Expression and miRNA Dysregulation in Individuals with 22q11.2 Deletion Syndrome
Source: PLoS One. 2014 Aug 1;9(8):e103884. doi: 10.1371/journal.pone.0103884 (PMC4118991; doi:10.1371/journal.pone.0103884)
Supplement: Table S1 — Fold copy number change by qPCR. (ΔKCt) for individuals with 22q11DS (n = 30) and TD individuals (n = 3) for three assays (PRODH, DGCR8, and D22S936) are shown. ΔKCt values for TUPLE1 and ZNF74 for one individual (#30) with an atypical deletion are shown to better characterize the deletion size. ΔKCt values of 0±0.35 indicate an equal ratio of the target and reference (normal) while values of −1±0.35 indicate a hemizygous deletion. (DOCX) [file pone.0103884.s001.docx]

**Table S1**

| **Number** | **Dx** | **PRODH** | **DGCR8** | **D22S936** | **TUPLE1** | **ZNF74** |
| --- | --- | --- | --- | --- | --- | --- |
| **1** | 22q | -1.48125 | -2.01225 | -0.83419 | - | - |
| **2** | 22q | -0.63652 | -0.95244 | -0.87660 | - | - |
| **3** | 22q | -0.64740 | -1.46973 | -0.71325 | - | - |
| **4** | 22q | -1.29535 | -1.16268 | -1.63264 | - | - |
| **5** | 22q | -0.91200 | -1.78136 | -0.64768 | - | - |
| **6** | 22q | -0.86621 | -1.43419 | -0.97246 | - | - |
| **7** | 22q | -1.39660 | -0.55266 | -0.48542 | - | - |
| **8** | 22q | -1.83660 | -0.62597 | -0.69816 | - | - |
| **9** | 22q | -0.89872 | -0.76361 | -0.82497 | - | - |
| **10** | 22q | -0.51701 | -0.49445 | -0.53526 | - | - |
| **11** | 22q | -1.04325 | -1.39731 | -1.03906 | - | - |
| **12** | 22q | -0.64740 | -0.56013 | -1.46973 | - | - |
| **13** | 22q | -1.20871 | -1.17939 | -0.56098 | - | - |
| **14** | 22q | -0.83343 | -0.91940 | -0.65776 | - | - |
| **15** | 22q | -0.94265 | -0.93989 | -0.56098 | - | - |
| **16** | 22q | -1.65794 | -0.58802 | -0.66171 | - | - |
| **17** | 22q | -1.61316 | -0.70300 | -1.25832 | - | - |
| **18** | 22q | -0.62922 | -1.61758 | -0.60579 | - | - |
| **19** | 22q | -0.90145 | -0.50127 | -0.80991 | - | - |
| **20** | 22q | -1.00280 | -1.79476 | -1.81431 | - | - |
| **21** | 22q | -0.55463 | -1.74704 | 0.59484 | - | - |
| **22** | 22q | -0.69161 | -1.10046 | -0.48424 | - | - |
| **23** | 22q | -1.62116 | -1.48394 | -1.61465 | - | - |
| **24** | 22q | -0.63851 | -0.89897 | -0.53369 | - | - |
| **25** | 22q | -0.49898 | -0.71936 | -0.58660 | - | - |
| **26** | 22q | -0.69555 | -1.54450 | -0.59927 | - | - |
| **27** | 22q | -0.74564 | -1.06611 | -0.87423 | - | - |
| **28** | 22q | -0.61533 | -0.51871 | -0.65681 | - | - |
| **29** | 22q | -1.43322 | -1.65144 | -1.42746 | - | - |
| **30** | 22q | 0.34307 | -0.72202 | 2.27022 | -1.01571 | -1.66769 |
| **Number** | **Dx** | **PRODH** | **DGCR8** | **D22S936** |  |  |
| 1 | TD | -0.14504 | 0.41664 | -0.46637 |  |  |
| 2 | TD | 0.24839 | 0.12468 | 0.00474 |  |  |
| 3 | TD | -0.33641 | -0.22891 | -0.48709 |  |  |
